# A Rare Lower Extremity Intramuscular Lipoma With Heterotopic Ossification

**DOI:** 10.1155/cro/2910875

**Published:** 2025-11-27

**Authors:** Ahmad Hammad, Ramy Saade, Hicham Moukaddam, Bruna Bacha, Mahmoud Hammad, Said Saghieh

**Affiliations:** ^1^Department of Orthopedics Surgery, American University of Beirut Medical Center, Beirut, Lebanon; ^2^Department of Diagnostic Radiology, American University of Beirut Medical Center, Beirut, Lebanon; ^3^Department of Radiology, University of Balamand, Balamand, Lebanon

**Keywords:** intramuscular lipoma, heterotopic ossification, lipoma, liposarcoma, soft tissue tumor

## Abstract

**Background:**

Most lipomatous soft tissue tumors are benign with a low risk of metastasis. Intramuscular lipoma is a very rare subtype with a very high rate of recurrence. When detected late, the differentiation between intramuscular lipoma and liposarcoma on imaging remains challenging.

**Case Presentation:**

A 49-year-old male presented with a lipomatous, radiographically lucent, soft tissue lesion in the lower extremity causing occasional numbness and tingling and associated with intralesional calcifications on MRI. The patient underwent a marginal excision of the lesion, which relieved his symptoms. Pathology revealed a lipoma with heterotopic ossification.

**Conclusions:**

Intramuscular lipomas are rare and underreported in the literature. When it is difficult to differentiate a lipoma from malignant liposarcoma, surgical intervention may be necessary. Long-term serial follow-ups are needed to assess for future recurrence.

## 1. Introduction

Soft tissue tumors (STTs) are a type of mesenchymal tumor that includes lipomatous tumors, fibrous and fibrohistiocytic tumors, and nerve sheath and vascular tumors [1]. In adults, lipomatous tumors are the second most common subgroup of tumors with a peak incidence between the fifth and seventh decades [1, 2].

Adipocytic tumors range from benign fat-containing masses to highly malignant liposarcomas (LPSs). Benign adipocytic tumors include lipoma, lipoblastoma, myolipoma, chondroid lipoma, spindle cell lipoma, lipomatosis, and hibernoma. Most lipomatous STTs are benign, whereas soft tissue sarcomas are fairly rare tumors, with an incidence of 4.7/100,000 per capita [3]. Benign lipomas have minimal risk of metastasis and recurrence. LPSs, on the contrary, are malignant with significant potential for metastasis and recurrence [4].

Intramuscular (im) lipoma is a very rare subtype that is located within muscle fibers and can infiltrate muscle or the synovium [5]. The local recurrence of the latter subtype is high reaching between 50% and 80%, whereas other lipomas have a 5% risk of local recurrence following marginal excision [1, 5, 6]. Most im lipomas of the extremity are detected late when the lesion becomes large enough to be palpable or to cause a mass effect on surrounding structures. Hence, a differential between im lipoma and LPS remains difficult despite imaging modalities and additional core biopsies, hence necessitating surgical resection.

There is not enough clinical data on case reports or treatment guidelines for cases of im lipomas. The case at hand presents a case of im lipoma with intralesional calcifications requiring biopsy and surgical excision for symptomatic relief and to rule out LPS. Both oral and written informed consents were obtained regarding the case study and future publications.

## 2. Case Presentation

A 49-year-old male presented with known diabetes mellitus, hypertension, and dyslipidemia. Six weeks prior to presentation to our clinic, the patient had originally presented to the emergency department after noticing his right calf to be swollen and tense with some paresthesia distally. A deep vein thrombosis was suspected and ruled out after performing a lower extremity venous duplex. Upon presentation to the orthopedic surgery clinics, the patient did not have pain, erythema, or hotness at the calf but reported only occasional numbness and tingling in the lower limb. On physical examination, he had a significant bulge in the right calf below the knee posterolaterally, that was mobile; he had palpable pulses, but no limitation in ambulation or range of motion prior to surgery.

**Figure 1:**
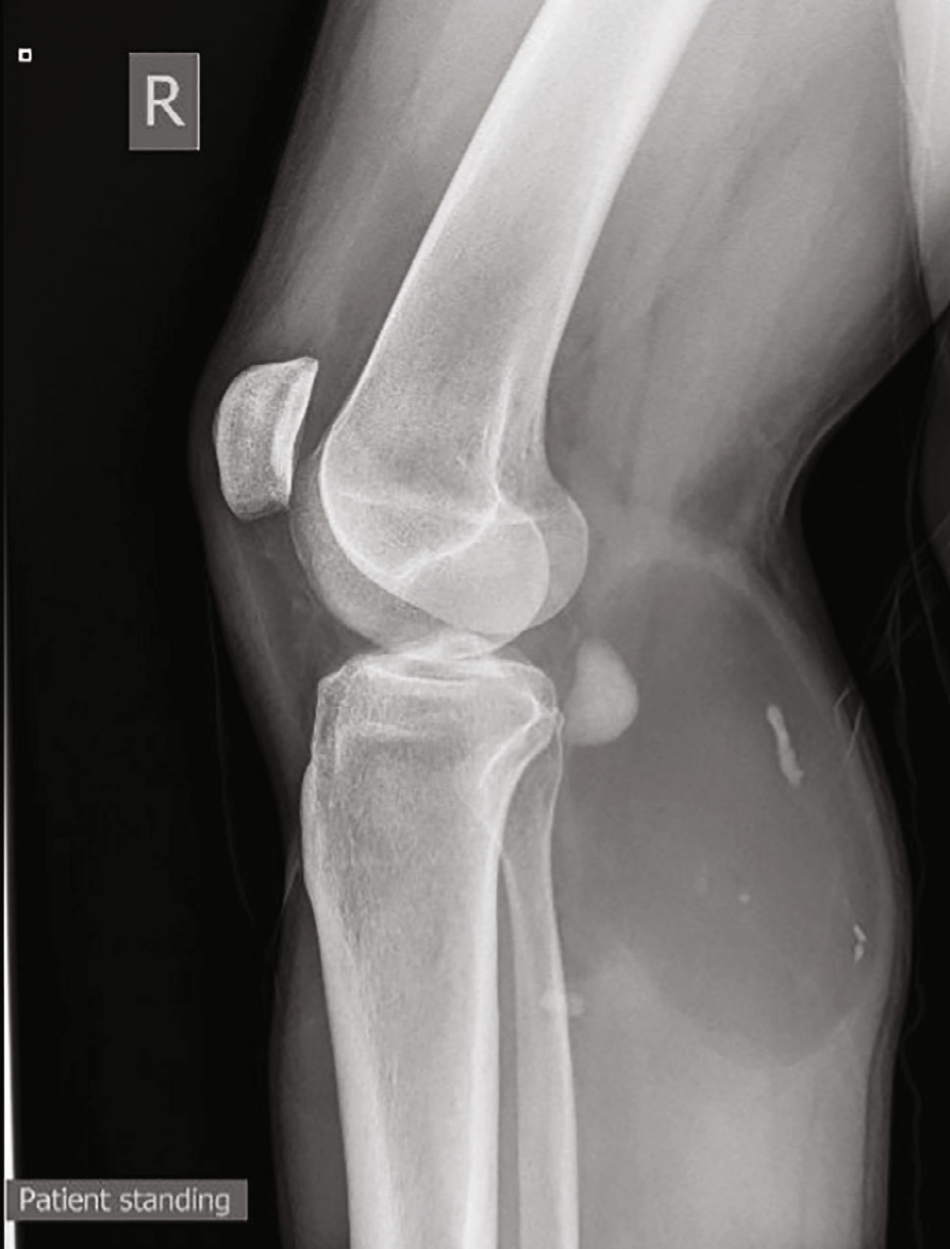
X-ray of the right leg showing large well-circumscribed ovoid lucent mass containing scattered calcifications, measuring 12 × 6.5 cm, in the posterior compartment of the tibia and fibula interface, suggestive of a lipoma.

**Figure 2:**
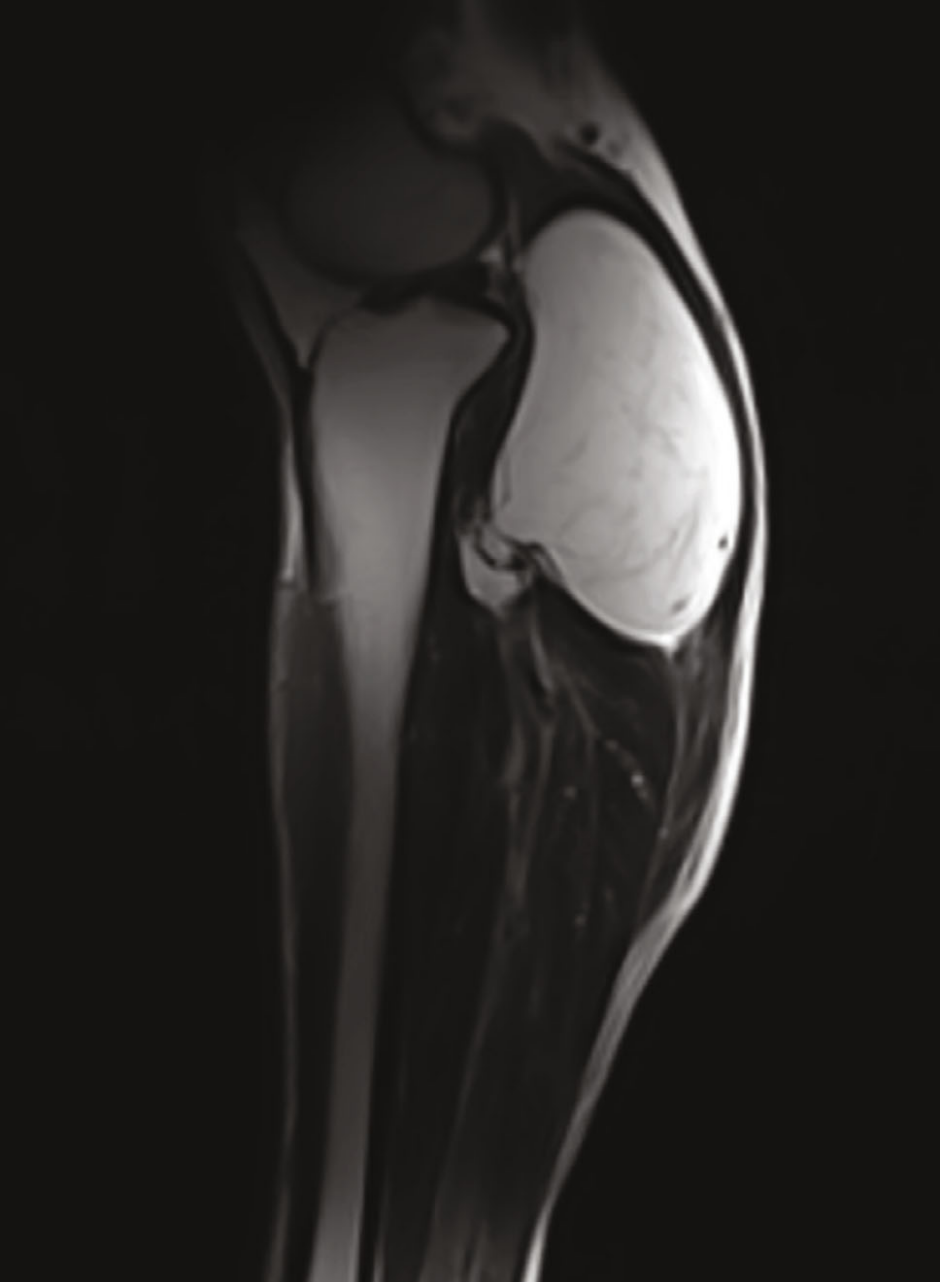
Sagittal view of the MRI of the right leg showing a soft tissue lipomatous lesion between the gastrocnemius and soleus reaching the posterior tibialis anteriorly without bony involvement or periosteal reaction.

**Figure 3:**
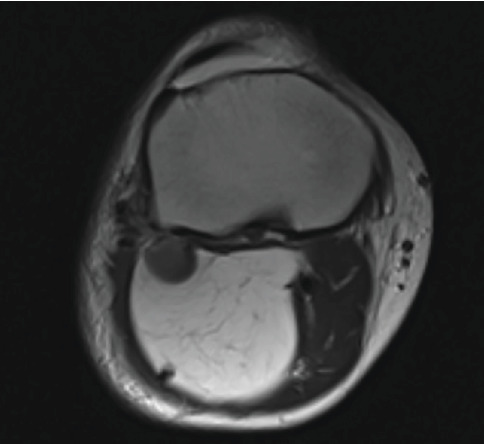
Axial view of the MRI of the right leg showing multiple intralesional calcifications and no septations.

Investigation was initiated to identify the underlying lesion. An x-ray of the leg showed a large well-circumscribed ovoid lucent mass containing scattered calcifications, measuring 12 × 6.5 cm, in the posterior compartment of the tibia and fibula interface, suggestive of a lipoma (Figure 1). MRI followed showing again a proximal deep calf lipomatous tumor within the gastrocnemius muscle and extending to reach the posterior tibialis muscle sheath (Figure 2). The lesion appears to be confined to the soft tissue and is not extending to the bone, with no periosteal reaction. There are also multiple calcifications within the lesion, but no septations; the most prominent seen in the posterior aspect of the tibiofibular interface (Figure 3). In the context of the presence of intralesional calcifications and given the patient's symptoms, he was referred for a core biopsy that showed ossifying lipoma.

Discussion between the medical team and the patient/family yielded a decision to proceed with marginal resection of the lipomatous lesion due to persistent neurological symptoms and the radiologic concern for LPS, although the core biopsy indicated a benign ossifying lipoma. Following a lazy-S incision at the posterior knee reaching the mid posterior leg, the lipoma was identified between the heads of the gastrocnemius and soleus. The common peroneal nerve, sural nerve, and posterior tibial tunnel were protected. The tumor was found reaching the posterior tibialis muscle and plantaris and was resected en bloc; then, common peroneal nerve decompression was performed.

Postoperatively, the patient had improvement in his neurologic symptoms, was pain-free, and was ambulatory without assistance. Pathology using hematoxylin and eosin staining revealed mature adipocytes admixed with well-formed trabecular bone and cartilage without atypia or mitotic activity, confirming benign lipoma with heterotopic ossification. Hence, no further surgical interventions or chemoradiotherapy were deemed necessary. Nevertheless, given the high rate of recurrence of im lipomas, frequent and long-term follow-up is required to assess for possible recurrence. At 6 months and at 12 months, the patient remained asymptomatic and recurrence-free, with full return to baseline function with preserved range of motion.

## 3. Discussion

We report a case of a rare im lipoma of the lower extremity associated with intralesional heterotopic ossification seen on imaging that required surgical resection to rule out LPS. This report adds to the limited available literature on im lipoma, and that despite its rare occurrence, clinicians should be alert in the proper clinical settings due to the high risk of recurrence even after surgery and to rule out LPS.

Clinically, lipomatous lesions can range from an asymptomatic mass seen on imaging or felt on examination to a painful tumor with accompanying symptoms related to mass effect and compressing surrounding neurovascular structures [2, 7]. im lipomas generally appear in sizes larger than 5 cm and are located below the deep subfascia of large muscle groups [8]. In the cases of incidental findings of lipomas or small asymptomatic benign lipomas, conservative nonsurgical intervention with observation is an option. However, surgical resection can be performed in cases of cosmesis or symptomatic lipomas secondary to mass effect. Additionally, in cases of im lipomas, wide excision with 1-cm margins might be needed to avoid the possibility of recurrence and to rule out a malignant LPS in the case of a large tumor [5].

Even though lipomas make up approximately half of all neoplasms within soft tissues, lipomas containing osseous elements are quite rare [9, 10]. Two proposed mechanisms are presented in the literature. The first suggests that ossifying lipomas result from the multidirectional differentiation of multipotent mesenchymal cells into both bone and adipose tissue [11]. The other mechanism is the metaplasia of fibrous elements into bone tissue, secondary to the existing “classic” lipoma in response to various external factors such as mechanical stress, repeated trauma, or ischemia; hence, the histological findings reveal disorganized lamellar bone adjacent to myxoid fibrous tissue and surrounded by uniform adipocytes [11, 12].

Radiologically, ossifying lipomas maintain a stable size and show maturing ossification over time, consistent with a long-standing indolent process. More aggressive tumors are more likely to contain thickened septa greater than 2 mm and nodular arrangements of nonadipose tissue representing internal dedifferentiated elements, whereas ossifying lipoma shows thin internal septa and lacks nodular enhancement [9]. More aggressive tumors are also more likely to have a larger overall size within a subfascial area of the body and consist of a lower composition of fatty tissue by percentage. Lipomas with no connection to bony structures showing osseous changes are rare [12].

It remains of paramount importance to differentiate between a benign lipomatous lesion and LPS. The main MRI characteristic that hints toward a malignant LPS is the broader and more nodular fibrous septa [3]. Sato et al. in their 10-year experience concluded that thick internal nonfatty septa are associated with LPS rather than a lipoma [13]. Histopathology shows multivacuolated lipoblasts, cellular pleomorphism, marked vascularization, and mitotic activity in the case of LPS [14]. The main problem is when an LPS is misdiagnosed as a benign lipoma, which bears extreme consequences, including aggressiveness, invasion of surrounding muscles neurovascular structures, and metastasis, resulting in poor outcomes. Therefore, and in aims to overcome the latter, it is recommended to biopsy all unclear and malignant tumors prior to resection. The controversy remains whether marginal excision or wide margin resection (> 1–2 cm) is necessary intraoperatively to avoid recurrence and reoperation [5].

This case highlights the diagnostic challenge of im lipoma with ossification mimicking LPS and reinforcing the role of surgical excision for both diagnosis and symptom relief. Recent in vivo animal studies have shown that near-infrared photodynamic therapy significantly diminished ectopic cartilage and subsequent bone formation, providing a new perspective for heterotopic ossification prophylaxis and treatment [15]. In addition, the notch signaling pathway as a genetic regulator when targeted and inhibited can lead to repressing ectopic cartilage and bone formation [16].

## 4. Conclusion

It remains challenging at times to differentiate a lipoma from malignant LPS when diagnostic modalities show intralesional features such as heterotopic ossification and septations, deeming surgical intervention necessary when the lesion becomes symptomatic with secondary mass effect. Despite the paucity of literature available on im lipoma and its rare incidence, clinicians must be attentive to identify such lesions in the proper clinical settings due to the high risk of recurrence even after surgical interventions and the need for long-term follow-ups.

## Data Availability

The data that support the findings of this study are available upon request from the corresponding author. The data are not publicly available due to privacy or ethical restrictions.
